# Effects of Planting Density and Nitrogen Fertilization on the Growth of Forage Rice in Reclaimed and General Paddy Fields

**DOI:** 10.3390/plants13010013

**Published:** 2023-12-19

**Authors:** Yeongmi Jang, Khulan Sharavdorj, Youngjik Ahn, Jinwoong Cho

**Affiliations:** 1 Department of Crop Science, College of Agricultural and Life Sciences, Chungnam National University, 99, Daehak-ro, Yuseong-gu, Daejeon 34134, Republic of Korea; yeongmi9202@naver.com (Y.J.); khulan1017@gmail.com (K.S.); 2 Department of Horticulture and Forestry, Pai Chai University, 155-40, Baejae-ro, Seo-gu, Daejeon 35345, Republic of Korea; yahn@pcu.ac.kr

**Keywords:** salinity, planting density, nitrogen fertilization, feed values, forage rice, soil condition

## Abstract

The purpose of this study is to identify the different effects exerted by planting density and nitrogen fertilization on high-salinity reclaimed paddy fields (RPF) and general paddy fields (GPFs), and to find the amount of fertilization and the planting density suitable for the growth of forage rice in each paddy field. Nitrogen fertilization with high-salt and low-salt soils, an untreated control plot, treatment with 200 kg/ha, 300 kg/ha, and 400 kg/ha, and planting densities of 30 cm × 10 cm and 30 cm × 16 cm, growth, and feed values were investigated. In both experimental locations, there was no significant change in the soil due to N treatment, but in the case of RPF, electrical conductivity (EC) decreased significantly from more than 5 dS/m to up to 2.87 dS/m during the yellow ripe stage due to the influence of floods and concentrated precipitation in the fields. In all soils, as both the amount of N treatment and the planting density increased, there was a proportional relationship in which the number of tillers and the dry weight also increased, with the occurrence of lodging also being increased. The dry weight, as expected, was 1.5 times higher at a planting distance of 10 cm, rather than 16 cm. In addition, in both locations, the N treatment led to an increase in the dry weight, but when N treatment reached 400 kg/ha (2.0), the dry weight decreased instead. Moreover, although there was no clear difference in feed value according to N treatment, in RPF, the neutral detergent fiber (NDF) was higher than 60%, the relative feed value (RFV) was less than 98, and the total digestible nutrient (TDN) was also low, confirming that the quality of rice was higher in GPF.

## 1. Introduction

A balanced fertilizer management of N, P, and K maintains or improves the N, P, and K balance, while the addition of residues further strengthens the nutrient balance [1]. The increase in the use of N fertilizers has caused the excessive use of nitrogen and pollution in some areas of the agricultural ecosystem since the 1950s, and the N surplus has since caused many problems for human and ecological health as it accumulates in the soil or disperses in the air, groundwater, and surface water through various pathways [2]. In addition, agricultural problems caused by a long-term use of substances such as pesticides and fertilizers were prominent, leading to not only contaminated agricultural products, but also problems in the soil, such as an imbalance in various nutrient ratios and the destruction of organic matter in the soil [3]. The long-term use of chemical fertilizers can impair the soil’s ability to sustain optimal crop growth and yield [4].

Furthermore, salinity is caused by the osmosis of salt in the soil solution, which produces the same effect as water stress caused by drought and may exert additional effects on crop growth; when an excessive amount of salt enters the plant, it rises to toxic levels in older transpiring leaves, causing premature senescence [5]. In general, sodium is not an essential factor in plant growth because plants do not have a special transport system for Na; if the plant is exposed to high Na concentrations, Na can move to plant cells through various channels [6]. Droughts, floods, heat waves, and the salinization of soils around the world are frequently occurring, affecting the production and cultivation of crops [7]. High salinity occurs for a variety of reasons, like dry soils, climate factors such as low precipitation and evaporation, coastal areas affected by tides, inadequate human irrigation, and the use of fertilizers and pesticides [8].

Already, the soil salt problem is increasing worldwide, which is a detrimental consequence of the ever-changing climate, and damaged soil quality and decreasing land areas are tasks to be solved [9]. In the case of Korea, the Korean plain accounts for less than 30–40% of the total area, most of which is covered with forests, and the proportion of land fit to cultivate agricultural products is low [10]. Therefore, the tidal mudflats in the West Sea were reclaimed to create arable land [11]; these areas were converted and utilized for various purposes such as industrial and urban development, power plants, and agriculture through the conversion of coastal wetlands with techniques such as reclamation and levees [12]. After the tidal mudflats are reclaimed, the soil salinity and vegetation in the area change rapidly, so land use planning is important because the soil in the reclaimed area is characterized by highly salinity [13]. Therefore, after 1950, the government focused on expanding farmland and cultivating highly productive rice varieties, effectively revitalizing the rural economy [14].

Therefore, in Korea, many experiments are being conducted on salt-resistant crops such as forage rice, beans, corn, and barley, etc. grown in rice paddies or field soils with a high salt content [15,16,17]. In line with this, since 1998, Korea has implemented a base-forage expansion program to encourage domestic forage production as a policy solution to reduce feed costs, which led to the expansion of the domestic forage production [18]. Various experiments are being conducted, including studies on the development and cultivation of varieties of feed rice in the general agricultural land, and intended to find the appropriate time for oats to be sown [19,20,21]. Furthermore, research has been actively conducted on aspects such as the productivity of forage crops according to the use of anaerobic liquid fertilizer [22], and the appropriate amount of fertilizer to be employed when cultivating the Barnyard millet for forage [23].

However, due to the nature of Korea’s climate, the rainy season in July and August can cause a significant amount of nutrient loss due to the outflow from the surface of rice paddies [24], so it can is necessary to find an appropriate level of chemical fertilizers because an excessive use of fertilizers lowers the water quality and leads to soil acidification [25,26]. In addition, there is a lack of experiments comparing the cultivation of feed rice in general agricultural land and reclaimed land, and there are also not many studies on the appropriate amount of fertilizer to be used in the cultivation of forage rice. Therefore, in this study, forage rice was grown in high-salt and low-salt paddy field soils, the objective of the present study being confirming changes in growth characteristics, yields, and feed value according to the different planting density and fertilizer treatments.

## 2. Results

### 2.1. Respective Field Conditions

**Figure 1:**
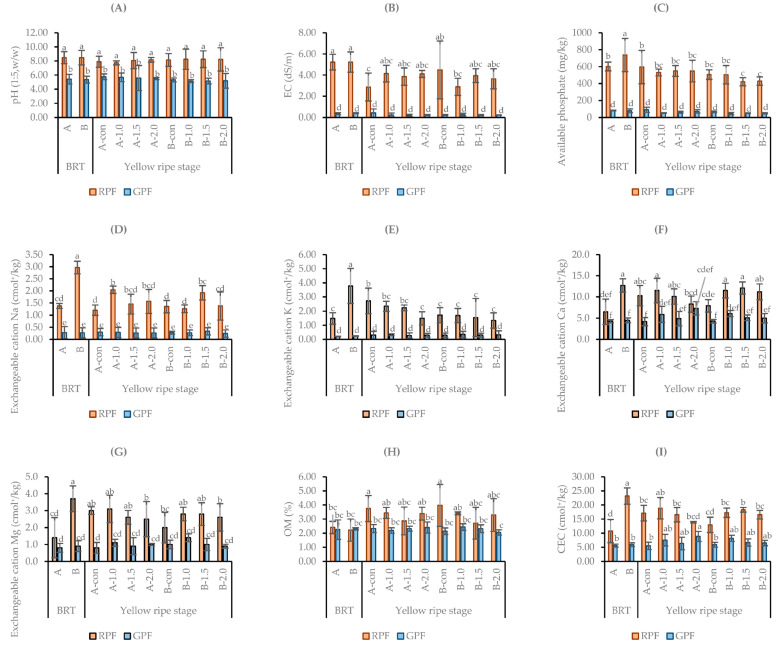
Analysis of soil before rice transplanting and at yellow ripe stage in reclaimed and general paddy fields. Abbreviations: RPF (reclaimed paddy field—Dangjin); GPF (general paddy field—Daejeon); BRT (before rice transplanting); planting density: A: 16 cm interval; B: 10 cm interval; fertilizer: control (0%); 1.0 (200 kg/ha); 1.5 (300 kg/ha); 2.0 (400 kg/ha); EC (electrical conductivity); OM (organic matter); CEC (cation exchange capacity); (**A**): pH, (**B**): EC, (**C**): AP, (**D**): Exchangeable cation Na, (**E**): Exchangeable cation K, (**F**): Exchangeable cation Ca, (**G**): Exchangeable cation Mg, (**H**): OM, (**I**): CEC; Lowercase letters from a to f express statistical results.

Figure 1 shows that the soils in the high-salinity reclaimed paddy field (RPF) and the low-salinity general paddy field (GPF) had different characteristics. Electrical conductivity (EC) is used as an indirect indicator of various physical and chemical properties of a soil [27]. In the two areas where the experiment was conducted, it was confirmed that the EC was lowered by constant floods: in particular, the high-salinity reclaimed paddy soil had an EC higher than 5 dS/m during the rice transplanting, but it decreased significantly to 2.87–4.49 dS/m during the yellow ripe stage.

Available phosphate was between 45–90 mg/kg in GPF, and it was substantially higher at over 422 mg/kg in RPF; additionally, in both locations, the levels slightly decreased compared to before rice transplanting, but there was no consistent trend according to the treatment plots. The organic matter was slightly higher within 2.05–3.97% in the reclaimed paddy soil, and increased in comparison to before rice transplanting, and similarly to available phosphate, there was no consistent trend according to the fertilizer treatment in both areas. The amount of exchangeable cation K in the reclaimed paddy soil was 1.34–2.72 cmol^+^/kg in all treatment plots, and the amount of exchangeable cation Ca was 7.9–12.1 cmol^+^/kg; both cations’ values increased after rice transplanting. The content of exchangeable cation Mg was 0.8–1.4 cmol^+^/kg in general paddy soils, but in reclaimed paddy soils, a 2.0–3.1 cmol^+^/kg Mg content was detected. The exchangeable cation Na amounted to 0.24–0.34 cmol^+^/kg in general paddy soils, while in reclaimed paddy soils, it amounted to a higher value of 1.21–2.97 cmol^+^/kg. The cation exchange capacity (CEC) is the total amount of cations adsorbed in a form that can be exchanged with other cations by electrical attraction in a certain amount of soil and at a specific pH. In this study, the CEC was 12.94–23.18 cmol^+^/kg in RPF, a value that was considerably higher than in GPF.

### 2.2. Growth and Development Characteristics

A two-way ANOVA statistical analysis was conducted to determine whether there was a significance between the location, the planting density, and the amount of fertilization in the data collected during the yellow ripe stage in the reclaimed and general paddy fields (Table 1).

Although the tiller number showed no significant difference depending on the location, but there was a significant difference within *p* < 0.001 in the planting density and fertilizer amount. Also, there was no interaction between the location, the planting density, and the amount of fertilizer. The tiller number and productive tiller in RPF were in the ranges of 193.6–488.4 and 127.6–468.6, respectively, while they were higher in GPF, in the range of 238.3–506.0 and 223.7–500.5, respectively. Moreover, both locations were found to be about 1.5–2 times more when the planting density was 10 cm compared to 16 cm. In addition, in both locations, when the amount of fertilizer was generally 1.5–2.0, there were higher tiller number and productive tiller, but it was confirmed that the degree of occurrence of lodging increased to 90% in RPF and 70% in GPF, when the planting density was 10 cm and the amount of fertilizer was 2.0.

It was confirmed that the plant height showed a significant difference (within *p <* 0.001) in between the location, the planting density, and the amount of fertilizer, but there was no interaction. In the case of the culm length, there was a significant difference within *p <* 0.01 depending on the planting density and fertilization amount, but similarly, it was shown that there was no interaction. The data show that the plant height was 159.8 cm–181.1 cm in RPF, while 139.5 cm–171.1 cm in GPF, with the RPF value being higher than expected. Although there were no consistent results depending on the treatment, the growth of crops untreated with fertilizers was not good when compared to other treatments. The culm length was the highest at 129.8 cm when the planting density was 10 cm and the fertilizer amount was 2.0 in RPF, but except for this, the highest culm length was 120.5 cm in RPF and 123.6 cm in GPF, when the fertilizer amount was set at 1.0 in both locations.

The chlorophyll content in the flag leaf interacted within *p <* 0.001 depending on the location, the planting density, and the amount of fertilizer, but in the third leaf, there was only a significant difference within *p <* 0.001 depending on the location, whilst there was no significance in the other groups. In general, it was confirmed that the chlorophyll content was greater when the planting density was 10 cm rather than 16 cm, with values in the range of 43.6–46.8 in RPF and 33.2–48.9 in GPF, and its content was higher in the flag leaf than in the third leaf. The flag leaf had the highest chlorophyll contents of 44.9 in RPF, when no fertilizer was applied and the planting density was 16 cm, and of 46.8, when the fertilizer amount was 1.0 and the planting density was 10 cm. Interestingly, the third leaf showed a common characteristic: both locations had a highest chlorophyll content of 46.8 and 43.4 in RPF and GPF, respectively, when the planting density was 16 cm and the fertilizer amount was 1.5, and of 46.6 and 42.2, in RPF and GPF, respectively, when the planting density was 10 cm and the fertilizer amount was 2.0. The leaf area index (LAI) showed a significant difference within *p <* 0.001, respectively, depending on the location, the planting density, and the amount of fertilizer, and an interaction also appeared within *p <* 0.001. The leaf area index was higher in GPF than in RPF, and both regions showed that the highest values were 4.8, 4.9 in RPF and 5.2 in GPF, respectively, when the fertilizer amount was 1.5, regardless of the planting density; however, exceptionally, in GPF, the highest leaf area index (9.6) was detected when e planting density was 10 cm and the amount of fertilizer was 2.0.

### 2.3. Chemical Analysis

Table 2 presents the data collected during the yellow ripe stage to find out the forage rice growth difference in the reclaimed and general paddy field according to the planting density and the amount of fertilizer. Crude protein (CP) interacted within *p <* 0.001 depending on the location, the planting density, and the amount of fertilizer. The CP content was higher in GPF than in RPF; in GPF, the CP content was the highest at 11.7 when the planting density was 16 cm and the fertilization amount was 2.0, and it was found to be the highest with a 2.0 fertilization amount in all treatment plots. 

Neutral detergent fiber (NDF) also showed an interaction within *p <* 0.01 depending on the location, the planting density, and the amount of fertilizer, but there was no significant difference in the acid detergent fiber (ADF) depending on the location; additionally, there was a significant difference within *p <* 0.05 in the planting density and fertilization amount, but there was no interaction. Both NDF and ADF were lower in GPF than in RPF, and in RPF, the amount of fertilizer was found to be the lowest in the untreated control plot. In addition, in GPF, at a planting density of 16 cm, NDF was the lowest at 53.9% in the untreated control plot, while ADF was the lowest at 31.8% when the fertilization amount was 2.0. Also, at a 10 cm planting density, both NDF and ADF were found to be the lowest at 43.4% and 28.2%, respectively, when the fertilization amount was 1.5.

**Table 2:** Rice feed values in reclaimed and general paddy fields during the yellow ripe stage.

Location	Planting Density	Fertilizer	CP	NDF	ADF	TDN	Hemicellulose	RFV
			(%)					
RPF	16							
		Control	9.7 bcd	59.7 d	37.0 abcd	59.7 abcde	22.7 abc	94 bc
		1.0	8.3 cde	61.2 d	36.1 abcd	60.4 abcde	25.1 ab	92 bc
		1.5	9.7 bcd	62.1 d	37.8 bcd	59.0 bcde	24.2 abc	89 bc
		2.0	9.9 bc	63.1 d	36.1 abcd	60.4 abcde	27.0 a	90 bc
	10							
		Control	7.3 ef	57.4 d	36.2 abcd	60.3 abcde	21.2 abc	98 bc
		1.0	10.0 b	60.5 d	36.4 abcd	60.1 abcde	24.0 abc	93 bc
		1.5	8.1 de	62.5 d	37.5 bcd	59.3 bcde	25.0 ab	89 bc
		2.0	10.6 ab	61.9 d	41.0 d	56.5 de	20.9 abc	86 bc
GPF	16							
		Control	4.9 g	53.9 bcd	36.4 abcd	60.2 abcde	17.6 abc	104 a
		1.0	6.2 fg	59.1 d	35.5 abcd	60.8 abcde	23.6 abc	96 bc
		1.5	10.6 ab	63.4 d	39.7 cd	57.6 cde	23.7 abc	85 bc
		2.0	11.7 a	55.2 cd	31.8 abc	63.8 abc	23.4 abc	108 a
	10							
		Control	5.6 g	46.6 abc	30.4 ab	64.9 ab	16.2 bc	130 a
		1.0	9.3 bcd	45.9 ab	32.0 abc	63.6 abcd	13.9 c	130 a
		1.5	8.3 cde	43.4 a	28.2 a	66.6 a	15.2 bc	144 a
		2.0	10.9 ab	63.6	43.2 d	54.7 e	20.3 abc	81 c
CV (%)			10.0	8.7	12.6	5.9	25.3	11.7
Location (L)			**	***	NS	NS	**	***
Planting density and fertilizer (P)			***	**	*	*	NS	***
L × P			***	**	NS	NS	NS	***

Abbreviations: RPF (reclaimed paddy field–Dangjin); GPF (general paddy field–Daejeon); Planting density: 16 cm interval; 10 cm interval; Fertilizer: control (0%); 1.0 (200 kg/ha); 1.5 (300 kg/ha); 2.0 (400 kg/ha); CP (crude protein); NDF (neutral detergent fiber); ADF (acid detergent fiber); TDN (total digestible nutrient); RFV (relative feed value).; ‘***’ *p* value < 0.001, ‘**’ *p* value < 0.01, ‘*’ *p* value < 0.05, ‘NS’ non-significant.; Lowercase letters from a to g express statistical results.

The total digestible nutrient (TDN) had no interaction based on location, planting density, and the amount of fertilizer, and no common phenomenon was observed according to the planting density and the amount of fertilization, but it was found to be the highest at 66.6% in GPF when the amount of fertilization was 1.5 in GPF. Hemicellulose showed no interaction depending on the location, the planting density, and the amount of fertilizer, but there were significant differences depending on the location, and it was confirmed that the content of hemicellulose was higher in RPF than in GPF. In RPF, at a planting density of 16 cm, hemicellulose was the highest at 27 when the amount of fertilizer was 2.0; at a planting density of 10 cm, hemicellulose was the highest at 25 when the amount of fertilizer was 1.5. In GPF, at a planting density of 16 cm, hemicellulose was the highest at 23.7 when the amount of fertilizer was 1.5; at a planting density of 10 cm, the hemicellulose was the highest at 20.3 when the amount of fertilizer was 2.0.

The relative feed value (RFV) interacted within *p <* 0.001 depending on the location, the planting density, and the amount of fertilizer, and in both locations, it was higher in GPF than RPF, also showing a higher content when the planting density was 10 cm rather than 16 cm. In addition, in RPF, RFV was the highest at 94 and 98, respectively, when the amount of fertilizer was an untreated control plot at all the planting densities. However, in GPF, at a planting density of 16 cm, RFV was 108 when the fertilizer amount was 2.0, but at a planting density of 10 cm, it was confirmed that RFV was the highest at 144 when the fertilizer amount was 1.5, but no consistent tendency was found according to the fertilizer amount.

### 2.4. Yield

**Figure 2:**
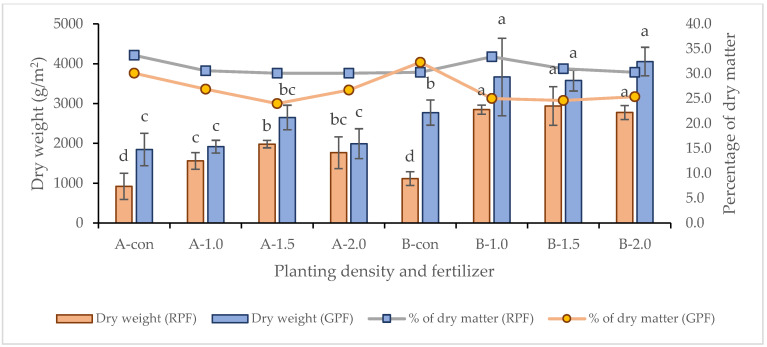
The dry weight and percentage of dry matter according to nitrogen fertilization and planting density in reclaimed and general paddy fields.; Abbreviations: RPF (reclaimed paddy field–Dangjin); GPF (general paddy field–Daejeon); Planting density: A: 16 cm interval; B: 10 cm interval; Fertilizer: control (0%); 1.0 (200 kg/ha); 1.5 (300 kg/ha); 2.0 (400 kg/ha).; Lowercase letters from a to d express statistical results.

Figure 2 shows the data related to dry weight and the percentage of dry matter in the reclaimed and general paddy field. 

In both areas, the fertilizer amount was the lowest in the untreated control plot, and in particular, the dry weight was the lowest at 922 g at a planting density of 10 cm with no fertilizer treatment. There was a clear difference between RPF and GPF: the dry weight was higher in GPF than in RPF, while in GPF, the percentage of dry matter was lower than in RPF. In RPF, with a 1.5 fertilizer amount and at planting densities of 10 cm and 16 cm, the dry weight was the highest at 1981 g/m^2^ and 2939 g/m^2^, respectively. In GPF, when the planting density was 16 cm and the fertilizer amount was 1.5, the dry weight was 2650 g, and when the planting density was 10 cm and the fertilizer amount was 2.0, the dry weight was the highest at 4055 g. However, when the fertilizer amount was 2.0, lodging occurred at the highest degree (Table 1); additionally, in GPF, except when the planting density was 10 cm, in all treatment plots, the dry weight was lower or not significantly different when the fertilizer amount was 2.0 rather than 1.0.

## 3. Discussion

If the application rate of the existing N fertilizer exceeds the capacity of vegetable uptake, the yield of the crop is significantly reduced, and a large amount of dissolved salt accumulates in the soil, which can affect secondary soil salinization and changes in soil salinity [28]. Also, it affects the germination, growth, and reproductive development of plants under salt stress conditions from the soil and ion toxicity and high osmotic stress cause nutrient deficiencies in plants that break the nutritional balance or interfere with nutrient (N, Ca, K, P, Fe, Zn) absorption, leading to a decrease in the agricultural productivity of crops. [7]. Therefore, through this experiment, nitrogen was applied to soil that suffered from salt stress and soil that did not—then the results were compared and analyzed, and the physiological response of the forage rice was evaluated to determine the appropriate amount of nitrogen fertilizer for each type of soil, as this whole process is necessary to ensure a stable production of grains.

When comparing GPF and RPF, a clear difference was observed between the two locations, as the soil pH, EC, AP, and the exchangeable cation Ca were all much higher in RPF (Figure 1). Studies have shown that the addition of NH_4_^+^-based N affected the net nitrification rates by changing the soil pH [29]; however, in this experiment, the addition of nitrogen fertilizer did not significantly change the pH in the soil. In the case of RPF, salt continuously rises from the subsurface soil [30], and it is assumed that the high pH was maintained because the water in the paddy field was drained at the time of the yellow ripe stage. According to a study by [31], EC in the soil is closely correlated with the exchangeable cation Na concentration, and the increase in Na concentration after waterlogging is caused by an increase in EC and soluble cation concentration, due to the dissolution of clay minerals including Na and insoluble salts. Also, it was found that the loss of exchangeable cation Ca and Mg from the soil exchange complex leads to soil acidification [32], and interestingly, our experimental results partly agree with this. In RPF, the contents of exchangeable cation Ca and Mg increased during the yellow ripe stage compared to the data measured before rice transplanting, and it was also confirmed that the pH in the soil was further alkalized to 7.74–8.25.

AP in the soil can be absorbed and used by plants, and in this experiment, it was observed in both RPF and GPF that, regardless of the planting density, the AP content was the highest in the untreated control plot. On the other hand, the yield was the lowest, and in the rest of the treatment plots—except for the untreated control plot—the lower the available phosphate, the higher the yield. A study by [33] also reported that high crop yields can be obtained by cultivation with an efficient P absorption, even in soils with a relatively low P content.

Looking at the changes in pH and EC in the soil, in the case of RPF with high salt, the pH was 8.47 before the rice transplanting, but slightly decreased to 7.74–8.25 during the yellow ripe stage, while the EC was 5.23 before the rice transplanting and decreased to 2.87–4.49 during the yellow ripe stage. According to [34], regardless of N application, the grain yield also decreases as EC increases at the same pH value or pH 8–9, but in this experiment, there was no effect on the untreated control plot, while in the remaining treatment plots, it was observed that a lower EC at a pH 8 in the soil generally leads to increased yields. Both pH and EC decreased after transplantation, although no consistent changes were detected according to the planting density or the fertilizer amount. Generally, studies have shown that although waterlogging cultivation can reduce salt in the root area, the effect of natural precipitation may be short [35]. Considering that another study showed that the ecosystem function of salt marshes has not been significantly affected by changes in precipitation for a year [36], likewise, in this experiment, it is estimated that the decrease in salt is a temporary effect due to waterlogging or concentrated precipitation.

**Figure 3:**
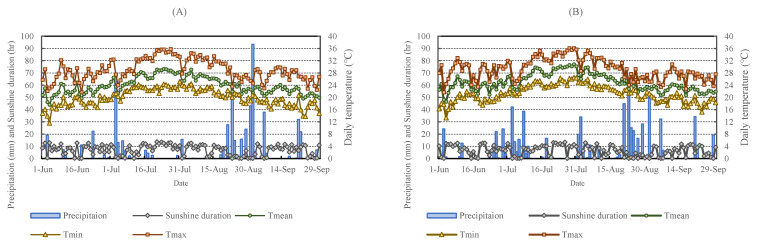
The average daily temperature (Tmean), minimum temperature (Tmin), maximum temperature (Tmax), and total precipitation and sunshine duration during the rice cultivation period of 2021: (**A**) Reclaimed paddy field (Dangjin); and (**B**) General paddy field (Daejeon).

Usually, the growth of plants is greatly affected by different water contents in the root area soil layer and the chemical components of the soil solutions under field conditions [37]. Therefore, even when the cultivar, planting density, and amount of fertilizer used are the same, differences in growth characteristics may appear. In GPF, the average temperature was 1 °C higher than in RPF, and the precipitation was also 91.8 mm higher in GPF than in RPF; however, the sunshine duration was 0.6 h longer in RPF (Figure 3). A common feature of this experiment is that the culm length height was generally similar regardless of the planting density in GPF and RPF, and it was observed that the plant height was about 10 cm higher in RPF (Table 1). In general, most cultivated varieties of crops show significantly decreased heights when exposed to salinity [38]. In addition, the salt stress reduces the uptake of nutrients such as N, P, K, Ca, etc., and it causes nutritional deficiencies in plants due to impaired transport efficiency [39]. However, in this experiment, the results were contrary to the expectations that the plant height is higher in high-salt soil than in low-salt soil, and there may be two causes for this. First, the height of the stem may have increased due to reduced salinity in the soil caused by additional precipitation, warming, and drought [35]. Secondly, it is presumed to be an overgrowth phenomenon in which the stems or leaves of plants grow long and tender. The reason for assuming that it was overgrowth is that, if the plant height increased in RPF, then a yield increase should have been noticeable; however, instead, the yield was higher in GPF. Likewise, the LAI was also higher in GPF, and in terms of feed value, TDN was also lower in RPF than in GPF. Usually, the causes for this phenomenon are the excessive application of fertilizer or excessive moisture, lack of sunshine, and abnormal temperatures. This was mostly the case during the course of this experiment, and it is thought that these factors influenced RPF more strongly than GPF.

Since the effect of N on the grain yields of early emerging tillers was limited, the further increase in yields thus mainly resulted from the contribution of late-emerging tillers [40]. Also, in the study of [41], growth parameters, plant height, LAI, the number of leaves, and DW, etc. were positively influenced by N treatment. In this experiment, it is confirmed that, in both locations, the N treatment affected the values of both the tiller number and LAI. The higher the planting density, the higher the number of tillers per unit area, and if N fertilizers are sufficiently applied, the number of rice tillers and panicles could increase [40]. In addition, the DM weight per square meter increases at the full heading stage with increasing planting density [42]. In this experiment, in both locations, the higher the planting density and the application of N, the higher the yield. However, lower yields were obtained in N treatment 2.0, which is judged to be due to the occurrence of lodging. When the N treatment was 2.0, the occurrence of lodging was 30–70% in GPF, but it was quite high at 80–90% in RPF (Table 1). In RPF, both the N treatment and the plant height were long, so it is believed that the occurrence of lodging increased due to complex causes. Lodging results in the primary effect of reducing canopy photosynthesis, rather than the potential of single leaf photosynthesis, which could lower the yields of grain [43].

According to a study by [44], at low salinity levels in soil, increasing the application rate of N fertilizers significantly improves the N uptake, thus alleviating the adverse effects caused by salinity; it is also said that high soil salinity acts as the dominating factor governing crop growth and N uptake. In addition, although the subsurface drainage of highly saline soil results in a loss of approximately 3–20% of the total applied nitrogen through the subsurface drainage effluent [45], another study showed that supplying an adequate amount of fertilizer depending on the size of the plant can produce high yields [46]. In this experiment, the yield in RPF was lower than that of GPF, but in RPF, the yield reached a level similar to that of an untreated control plot in GPF due to the application of N. This had a positive effect by contributing to the increase in growth and yield by N treatment (Figure 2). In general, this was consistent with several studies reporting that crop yields are greatly suppressed at high levels of salinity, but that applying an appropriate amount of N alleviates the negative effects of salinity on the yields [34,47]. Moreover, in RPF, the yield peaked with the application of N 1.5 for both the planting densities of 10 cm and 16 cm, while the linearly increased yield was not obtained with the application of N 2.0. Namely, Mitscherlich’s law of diminishing returns applies to this case, as it states that marginal productivity decreases as the level of marginal factors is increased [48]. The rice grain yield peaked at a nitrogen application rate of 300 kg/ha, and the supply of nitrogen above 300 kg/ha was highly consistent with the results of studies showing that it failed to ensure a linearly increased rice yield [49]. Usually, as the seeding rate increases, the availability of resources increases, and the forage yield increases. However, at the highest planting density, competition between plants intensifies, and similarly to the findings that forage yields did not respond to N application, this experiment also showed how the N treatment above 2.0 did not lead to increased yields.

In this experiment, the N treatment and planting density did not notably affect the feed value, but when comparing the value of forage in RPF and GPF, it was found to be better in GPF (Table 2). The results in this experiment are in agreement with the study by [50], which states that the forage quality indexes, including CP and ADF, were not affected by the interaction between N and the seeding rate. Nevertheless, in this study, CP was the highest at the N 2.0 treatment among all treatments, regardless of the planting density, but no other remarkable differences were observed. TDN and RFV, the most important factors in feed value assessment, did not show a consistent difference for the different N treatments and planting densities, and both were confirmed to be higher in GPF than in RPF. In GPF, TDN and RFV showed similar trends, and were the best when the planting density was 10 cm, N treatment was 2.0, the planting density was 16 cm, and the N treatment was 1.5. However, they were observed to be somewhat higher in an untreated control plot than in the other treatment plots in RPF.

## 4. Materials and Methods

### 4.1. Experimental Design

A joint experiment was conducted in two locations: a high-salinity paddy soil in the reclaimed land in Seokmun District, Gagok-ri, Songsan-myeon, Dangjin-si, Chungcheongnam-do (36°59′20.7 N, 126°40′10.6 E), and a low-salinity paddy soil in the farm belonging to Chungnam National University, 99, Daehak-ro, Yuseong-gu, Daejeon Metropolitan City (36°22′05.4 N, 127°21′13.3 E). Mogwoo, a type of forage rice used in this study, was provided by the National Institute of Crop Science; it is a late variety which can be cultivated even in high-salinity soil [51]. The rice seeds were disinfected by the hot water soaking method, then sowed on a seedbed on 10 May 2021, and grown for 30 days; then, rice transplanting was conducted in Daejeon (GPF) on 3 June and Dangjin (RPF) on 4 June, respectively. In both Dangjin and Daejeon, the experimental area was divided into 12 sections for a total of 24 sections, with each section measuring 5 m × 3.3 m. The experiment was conducted with three replicates for each of the four treatments. Nitrogen fertilization with “Luxury NK (N:200-P:80-K:80)” fertilizer was performed on four different treatment plots based on a previous experiment, where a 300 kg/ha fertilizer amount was used [49]. This study was designed as follows: an untreated control plot and treatments with three nitrogen rates, 200 kg/ha (1.0), 300 kg/ha (1.5), and 400 kg/ha (2.0). Furthermore, basal fertilization was 100% on 11 June, while the top dressing was 80% on both 16 July and 11 August. In both areas, the planting density was 30 cm × 10 cm and 30 cm × 16 cm, respectively, and 5–8 individuals were transplanted per hill. No pesticides, including herbicides, or weed removal methods were applied, and the rice and soil samples were collected during the yellow ripe stage, four weeks after the heading stage: then, sample collection was carried on in Dangjin on 30 September and Daejeon on 1 October.

### 4.2. Weather Conditions and Measurement of Soil Samples

Meteorological data measurement was carried on with the data provided by the Korea Meteorological Administration: the collected data only span the cultivation period in the reclaimed paddy field (Dangjin) and the general paddy field (Daejeon) (Figure 3). The total precipitation was 616.3 mm in RPF and 708.1 mm in GPF, and thus higher in GPF. Since GPF frequently had rainy days, the average duration of sunshine was 6.3 h, which was lower than that of RPF. In addition, the average temperature in RPF was 23.9 °C, while in GPF, it was 24.9 °C, about 1 °C higher than in RPF.

The soil samples were analyzed by collecting topsoil (about 0–10 cm from the surface) from each soil in three replicates. They were then let to dry naturally for a week and passed through a 2 mm sieve. The soil analysis was conducted in accordance with the Rural Development Administration soil and plant analysis method (NIAST, 2000). pH and EC values were measured with a pH meter (CP-500L, iSTEK, Seoul, Republic of Korea) and an EC meter (CON450, THERMO, Singapore) using soil and distilled water at a ratio of 1:5. The available phosphate (AP) was analyzed with the Lancaster method and the organic matter (OM) with the Tyurin method. Exchangeable cations Ca, K, Mg, and Na were extracted with 1N-NH_4_OAC (pH 7.0) and analyzed with an ICP (Varian Vista-MPX, Varian, Palo Alto, CA, USA). In addition, the cation exchange capacity (CEC) was analyzed with the 1N-ammonium acetate method.

### 4.3. Measurement of Growth Characteristics of Rice

The samples used to investigate the growth characteristics were collected in three replicates in the center of each plot: five plants were collected in each of the three 1 m^2^ central areas of all four plots. When the average planting distance was 10 cm, the density was 33 hills/m^2^, while when the average planting distance was 16 cm, the density resulted to be 22 hills/m^2^; values calculated for 22 hills and 33 hills were used to obtain the total tiller number and the productive tiller data, respectively. The tiller number and productive tiller data were collected manually while the plant height and culm length were measured with a 100 cm ruler. The portable chlorophyll meter (SPAD-502Plus, Konica Minolta Sensing Americas, Inc., Ramsey, NJ, USA) is a device that was used in this study to measure the chlorophyll content in the flag leaf and third leaf of rice in a non-destructive manner. The chlorophyll content was directly measured in rice paddies, then the samples were collected and a growth survey was conducted. The leaf area index (LAI) was measured using the LI-3100 Area Meter (Lincoln, NE, USA). In the case of lodging, 0 means that there are no plants that have fallen over, while the degree of fallen plants increases as the number approaches 100 (Table 1).

### 4.4. Chemical Analysis for Forage Quality

The samples were dried at 80 °C for 72 h following a method from a previous study [52] in order to minimize the feed value loss due to the use of a forced connection oven (LDO-150F); then, the dry weight was measured: subsequently, the samples, which were pulverized by combining all the leaves, stems, and grain samples, were used for analysis. Crude protein (CP) was analyzed using the Kjeldahl method according to the standard analysis method of AOAC (1995). Neutral detergent fiber (NDF) and acid detergent fiber (ADF) were evaluated with the method of Goering and van Soest (1970). Total digestible nutrient (TDN) was obtained with Holland and Kezar’s (1992) feed value evaluation formula and was calculated using Formula (1). The relative feed value (RFV) was calculated by the calculation formula of (2), and the percentage of dry matter was calculated by (3).

TDN (%) = 88.9 − (0.79 × ADF (%))(1)

RFV = DDM (%) × DMI (%)/1.29  [DDM (%) = 88.9 − (ADF(%) × 0.779), DMI(%) = 120/NDF(%)](2)

Percentage of dry matter (%) = (dry weight/fresh weight) × 100(3)

### 4.5. Statistical Analysis

R ver.4.1.2 (R Core Team, 2021) was used for the statistical analysis of all data, and a two-way ANOVA analysis was conducted to confirm the interaction according to the location, planting density, and fertilizer amount. The Duncan test (Duncan’s multiple range test) was performed as a post hoc test. It was tested within the significance level of *p <* 0.05, and all data were collected in at least three replicates.

## 5. Conclusions

In this study, in the soil, pH, EC, AP, and exchangeable cations Na, K, Ca, Mg, and CEC were all higher in RPF than in GPF. The higher the N application rates, the more severe the lodging, resulting in a decrease in yields. Such an effect was observed to have a greater impact on RPF than on GPF. In addition, it was confirmed that increasing the planting density along with the N treatment led to an increase in the tiller number and crop yield, while there was no noticeable change in the feed value. In RPF, the highest dry weights of 1981 g/m^2^ and 2939 g/m^2^ were obtained with a planting density of 10 cm and 16 cm, respectively, when the N treatment was 1.5. In GPF, the highest dry weight was 2650 g/m^2^ at N 1.5 treatment and at a planting density of 16 cm, and it also was the highest at 4055 g/m^2^ at N 2.0 treatment and at a planting density of 10 cm; however, it was judged to be inappropriate because of the high degree of lodging. For this reason, it is considered most appropriate to apply the N 1.0 treatment, so that a dry weight as high as 3666 g/m^2^ can be ensured and the lodging can be minimized. Therefore, through this experiment, we highlighted the need for further research to find the cause of the low correlation between the N treatment, soil, and feed value, and to explore ways to increase feed value in RPF.

## Figures and Tables

**Table 1:** The growth and development characteristics of rice in reclaimed and general paddy fields during the yellow ripe stage.

Location	Planting Density	Fertilizer	Tiller Number	Productive Tiller	Plant Height	Culm Length	SPAD		LAI (m^2^)	Lodging
							Flag Leaf	Third Leaf		
			(No./m^2^)		(cm)					0–100%
RPF	16									
		Control	193.6 f	127.6 f	160.0 cd	106.6 c	44.9 abc	44.2 ab	2.7 e	0
		1.0	255.2 ef	233.2 ef	180.7 a	120.9 abc	44.4 abc	43.4 ab	3.2 cde	0
		1.5	321.2 cdef	167.2 cdef	172.2 abc	115.5 abc	44.8 abc	46.8 a	4.8 bcd	10
		2.0	294.8 def	286.0 def	168.5 abc	116.3 abc	40.6 c	43.5 ab	2.3 e	80
	10									
		Control	310.2 cdef	303.6 cdef	159.8 cd	112.6 bc	45.6 abc	44.1 ab	2.5 e	0
		1.0	455.4 ab	415.8 ab	177.0 ab	120.5 abc	46.8 abc	43.3 ab	4.7 bcd	10
		1.5	488.4 a	468.6 a	171.0 abc	119.3 abc	43.6 abc	43.5 ab	4.9 bcd	20
		2.0	429.0 abc	409.2 abc	181.1 a	129.8 a	43.8 abc	46.6 a	3.8 cde	90
GPF	16									
		Control	238.3 ef	231.0 ef	148.0 de	112.2 bc	31.3 d	27.3 d	2.9 de	0
		1.0	245.7 ef	238.3 ef	164.9 bc	123.0 ab	41.7 bc	38.0 bc	3.5 cde	0
		1.5	348.3 bcde	341.0 bcde	167.0 abc	118.7 abc	47.3 ab	43.4 ab	5.2 bc	10
		2.0	264.0 ef	223.7 ef	171.1 abc	114.0 bc	46.5 abc	41.1 abc	4.2 cde	30
	10									
		Control	401.5 abcd	401.5 abcd	139.5 e	107.0 c	33.2 d	33.9 cd	4.9 bcd	0
		1.0	500.5 a	489.5 a	166.4 abc	123.6 ab	44.7 abc	38.0 bc	6.5 b	0
		1.5	495.0 a	484.0 a	168.3 abc	117.4 abc	48.9 a	40.6 abc	9.3 a	20
		2.0	506.0 a	500.5 a	158.7 cd	111.5 bc	44.5 abc	42.2 ab	9.6 a	70
CV (%)			21.5	23.8	5.5	7.2	8.3	11.4	26.6	-
Location (L)			NS	**	***	NS	*	***	***	-
Planting density and fertilizer (P)			***	***	***	**	**	NS	***	-
L × P			NS	NS	NS	NS	***	NS	***	-

Abbreviations: RPF (reclaimed paddy field–Dangjin); GPF (general paddy field–Daejeon); Planting density: 16 cm interval; 10 cm interval; fertilizer: control (0%); 1.0 (200 kg/ha); 1.5 (300 kg/ha); 2.0 (400 kg/ha); LAI (leaf area index).; ‘***’ *p* value < 0.001, ‘**’ *p* value < 0.01, ‘*’ *p* value < 0.05, ‘NS’ non-significant.; Lowercase letters from a to f express statistical results.

## Data Availability

Not applicable.

## References

[1] Linquist B.A., Phengsouvanna V., Sengxue P. (2007). Benefits of organic residues and chemical fertilizer to productivity of rain-fed lowland rice and to soil nutrient balances. Nutr. Cycl. Agroecosyst..

[2] Eickhout B., Bouwman A.F., van Zeijts H. (2006). The role of nitrogen in world food production and environmental sustainability. Agric. Ecosyst. Environ..

[3] Wang J., Li R., Zhang H., Wei G., Li Z. (2020). Beneficial bacteria activate nutrients and promote wheat growth under conditions of reduced fertilizer application. BMC Microbiol..

[4] Xing Y., Wang C., Li Z., Chen J., Li Y. (2023). Effect and Mechanism of Rice-Pasture Rotation Systems on Yield Increase and Runoff Reduction under Different Fertilizer Treatments. Agronomy.

[5] Munns R. (2002). Comparative physiology of salt and water stress. Plant. Cell Environ..

[6] Shahid M.A., Sarkhosh A., Khan N., Balal R.M., Ali S., Rossi L., Gómez C., Mattson N., Nasim W., Garcia-Sanchez F. (2020). Insights into the physiological and biochemical impacts of salt stress on plant growth and development. Agronomy.

[7] Shrivastava P., Kumar R. (2015). Soil salinity: A serious environmental issue and plant growth promoting bacteria as one of the tools for its alleviation. Saudi J. Biol. Sci..

[8] Yadav S., Irfan M.D., Ahmad A., Hayat S. (2011). Causes of salinity and plant manifestations to salt stress: A review. J. Environ. Biol..

[9] Kumawat K.C., Sharma B., Nagpal S., Kumar A., Tiwari S., Nair R.M. (2023). Plant growth-promoting rhizobacteria: Salt stress alleviators to improve crop productivity for sustainable agriculture development. Front. Plant Sci..

[10] Lee C.H., Lee B.Y., Chang W.K., Hong S., Song S.J., Park J., Kwon B.O., Khim J.S. (2014). Environmental and ecological effects of Lake Shihwa reclamation project in South Korea: A review. Ocean Coast. Manag..

[11] Kim S.H., Kim K., Lee M., Jeong H.J., Kim W.J., Park J.G., Yang J.S. (2009). Enhanced benthic nutrient flux during monsoon periods in a coastal lake formed by tideland reclamation. Estuaries Coasts.

[12] Kim S.G. (2010). The evolution of coastal wetland policy in developed countries and Korea. Ocean Coast. Manag..

[13] Cho K.H., Beon M.S., Jeong J.C. (2018). Dynamics of soil salinity and vegetation in a reclaimed area in Saemangeum, Republic of Korea. Geoderma.

[14] Choi Y.R. (2014). Modernization, Development and Underdevelopment: Reclamation of Korean tidal flats, 1950s–2000s. Ocean Coast. Manag..

[15] Jang Y., Sharavdorj K., Nadalin P., Lee S., Cho J. (2022). Growth and Forage Value of Two Forage Rice Cultivars According to Harvest Time in Reclaimed Land of South Korea. Agronomy.

[16] Sohn Y.-M., Jeon G.-Y., Song J.-D., Lee J.-H., Park M.-E. (2009). Effect of soil salinity variation on the growth of barley, rye and oat seeded at the newly reclaimed tidal lands in Korea. Korean J. Soil Sci. Fertil..

[17] Lee S., Bae H.-S., Lee S.-H., Kang J.-G., Kim H.-K., Lee K.-B., Park K.-H. (2013). Effect of Soil Salinity Levels on Silage Barley Growth at Saemangeum Reclaimed Tidal Land. Korean J. Soil Sci. Fertil..

[18] Chang J.B. (2018). The effects of forage policy on feed costs in Korea. Agriculture.

[19] Ahn E., Won Y., Kang K., Park H., Jung K., Hyun U., Lee Y. (2022). Feed Value of the Different Plant Parts of Main Forage Rice Varieties. Korean J. Crop Sci..

[20] Jung J.-S., Lee B.-H., Choi B.-R., Han O.-K., Park H.-S., Choi K.-C. (2023). Study on the Forage Cropping System Linked to Forage Barley, Whole Crop Rice and Barnyard Millet at Paddy Fields in Cheonan Region, South Korea. J. Korea Acad. Coop. Soc..

[21] Park J., Kim Y., Yoon Y., Choi S., Park J., Park H. (2022). An Optimum Summer Cultivation Sowing Date for Seed Production of Oats (*Avena sativa* L.). Korean J. Crop Sci..

[22] Yoon Y.-M., Shin K.-S., Hwang W.-J., Lee S.-H., Kim C.-H. (2012). Nutrient Value and Yield Response of Forage Crop Cultivated in Reclaimed Tidal Land Soil Using Anaerobic Liquid Fertilizer. Korean J. Org. Agric..

[23] Hwang J.-B., Park T.-S., Park H.-K., Kim H.-S., Choi I.-B., Bae H.-S. (2017). Effect of Seeding and Nitrogen rates on the Growth characters, Forage yield, and Feed value of Barnyard millet in the Reclaimed tidal land. Weed Turfgrass Sci..

[24] Cho J.Y., Son J.G., Choi J.K., Song C.H., Chung B.Y. (2008). Surface and subsurface losses of N and P from salt-affected rice paddy fields of Saemangeum reclaimed land in South Korea. Paddy Water Environ..

[25] Conant R.T., Berdanier A.B., Grace P.R. (2013). Patterns and trends in nitrogen use and nitrogen recovery efficiency in world agriculture. Glob. Biogeochem. Cycles.

[26] Zeng M., De Vries W., Bonten L.T.C., Zhu Q., Hao T., Liu X., Xu M., Shi X., Zhang F., Shen J. (2017). Model-Based Analysis of the Long-Term Effects of Fertilization Management on Cropland Soil Acidification. Environ. Sci. Technol..

[27] Sudduth K.A., Kitchen N.R., Wiebold W.J., Batchelor W.D., Bollero G.A., Bullock D.G., Clay D.E., Palm H.L., Pierce F.J., Schuler R.T. (2005). Relating apparent electrical conductivity to soil properties across the north-central USA. Comput. Electron. Agric..

[28] Han J., Shi J., Zeng L., Xu J., Wu L. (2015). Effects of nitrogen fertilization on the acidity and salinity of greenhouse soils. Environ. Sci. Pollut. Res..

[29] Wang J., Tu X., Zhang H., Cui J., Ni K., Chen J., Cheng Y., Zhang J., Chang S.X. (2020). Effects of ammonium-based nitrogen addition on soil nitrification and nitrogen gas emissions depend on fertilizer-induced changes in pH in a tea plantation soil. Sci. Total Environ..

[30] Qadir M., Oster J.D. (2004). Crop and irrigation management strategies for saline-sodic soils and waters aimed at environmentally sustainable agriculture. Sci. Total Environ..

[31] Hemati Matin N., Jalali M. (2017). The effect of waterlogging on electrochemical properties and soluble nutrients in paddy soils. Paddy Water Environ..

[32] Brinkman R. (1970). Ferrolysis, a hydromorphic soil forming process. Geoderma.

[33] Balemi T., Negisho K. (2012). Management of soil phosphorus and plant adaptation mechanisms to phosphorus stress for sustainable crop production: A review. J. Soil Sci. Plant Nutr..

[34] Huang L., Liu X., Wang Z., Liang Z., Wang M., Liu M., Suarez D.L. (2017). Interactive effects of pH, EC and nitrogen on yields and nutrient absorption of rice (*Oryza sativa* L.). Agric. Water Manag..

[35] Charles H., Dukes J.S. (2009). Effects of warming and altered precipitation on plant and nutrient dynamics of a New England salt marsh. Ecol. Appl..

[36] Emery H.E., Angell J.H., Fulweiler R.W. (2019). Salt Marsh Greenhouse Gas Fluxes and Microbial Communities Are Not Sensitive to the First Year of Precipitation Change. J. Geophys. Res. Biogeosci..

[37] Rengasamy P., De Lacerda C.F., Gheyi H.R. (2022). Salinity, Sodicity and Alkalinity. Subsoil Constraints for Crop Production.

[38] Puvanitha S., Mahendran S. (2017). Effect of salinity on plant height, shoot and root dry weight of selected rice cultivars. Sch. J. Agric. Vet. Sci..

[39] Razzaq A., Ali A., Safdar L.B., Zafar M.M., Rui Y., Shakeel A., Shaukat A., Ashraf M., Gong W., Yuan Y. (2020). Salt stress induces physiochemical alterations in rice grain composition and quality. J. Food Sci..

[40] Wang Y., Ren T., Lu J., Ming R., Li P., Hussain S., Cong R., Li X. (2016). Heterogeneity in rice tillers yield associated with tillers formation and nitrogen fertilizer. Agron. J..

[41] Elsiddig A.M.I., Zhou G., Zhu G., Nimir N.E.A., Suliman M.S.E., Ibrahim M.E.H., Ali A.Y.A. (2023). Nitrogen fertilizer promoting salt tolerance of two sorghum varieties under different salt compositions. Chil. J. Agric. Res..

[42] Nakano H., Morita S., Kitagawa H., Wada H., Takahashi M. (2012). Grain Yield Response to Planting Density in Forage Rice with a Large Number of Spikelets. Crop Sci..

[43] Setter T.L., Laureles E.V., Mazaredo A.M. (1997). Lodging reduces yield of rice by self-shading and reductions in canopy photosynthesis. F. Crop. Res..

[44] Chen W., Hou Z., Wu L., Liang Y., Wei C. (2010). Effects of salinity and nitrogen on cotton growth in arid environment. Plant Soil.

[45] Singh M., Bhattacharya A.K., Nair T.V.R., Singh A.K. (2002). Nitrogen loss through subsurface drainage effluent in coastal rice field from India. Agric. Water Manag..

[46] Villa-Castorena M., Ulery A.L., Catalán-Valencia E.A., Remmenga M.D. (2003). Salinity and Nitrogen Rate Effects on the Growth and Yield of Chile Pepper Plants. Soil Sci. Soc. Am. J..

[47] Ibrahim M.E.H., Zhu X., Zhou G., Ali A.Y.A., Ahmad I., Elsiddig A.M.I. (2018). Nitrogen fertilizer reduces the impact of sodium chloride on wheat yield. Agron. J..

[48] Ferreira I.E.P., Zocchi S.S., Baron D. (2017). Reconciling the Mitscherlich’s law of diminishing returns with Liebig’s law of the minimum. Some results on crop modeling. Math. Biosci..

[49] Chen Y., Liu Y., Ge J., Li R., Zhang R., Zhang Y., Huo Z., Xu K., Wei H., Dai Q. (2022). Improved physiological and morphological traits of root synergistically enhanced salinity tolerance in rice under appropriate nitrogen application rate. Front. Plant Sci..

[50] Hajighasemi S., Keshavarz-Afshar R., Chaichi M.R. (2016). Nitrogen fertilizer and seeding rate influence on grain and forage yield of dual-purpose barley. Agron. J..

[51] Lee S.-B., Yang C.-I., Lee J.-H., Kim M.-K., Shin Y.-S., Lee K.-S., Choi Y.-H., Jeong O.-Y., Jeon Y.-H., Hong H.-C. (2013). A Late-Maturing and Whole Crop Silage Rice Cultivar “Mogwoo”. J. Korean Soc. Grassl. Forage Sci..

[52] Padmathilake K.R.E., Wickramaarachchi V.N., Anver M.A.M.S., Bandara D.C. (2007). Biological and economical feasibility of growing mint (*Mentha sylvestris*), mustard (*Brassica integrifolia*) and asamodagam (*Trachyspermum involucratum*) under hydroponics. Trop. Agric. Res..

